# Electrocardiographic diagnosis of left ventricular hypertrophy in aortic valve disease: evaluation of ECG criteria by cardiovascular magnetic resonance

**DOI:** 10.1186/1532-429X-11-18

**Published:** 2009-06-01

**Authors:** Stefan Buchner, Kurt Debl, Josef Haimerl, Behrus Djavidani, Florian Poschenrieder, Stefan Feuerbach, Guenter AJ Riegger, Andreas Luchner

**Affiliations:** 1 Klinik und Poliklinik für Innere Medizin II, Universitätsklinikum Regensburg, Germany; 2 Institut für Röntgendiagnostik, Universitätsklinikum Regensburg, Germany; 3 Medizinische Klinik, Klinikum Landshut Achdorf, Germany

## Abstract

**Background:**

Left ventricular hypertrophy (LVH) is a hallmark of chronic pressure or volume overload of the left ventricle and is associated with risk of cardiovascular morbidity and mortality. The purpose was to evaluate different electrocardiographic criteria for LVH as determined by cardiovascular magnetic resonance (CMR). Additionally, the effects of concentric and eccentric LVH on depolarization and repolarization were assessed.

**Methods:**

120 patients with aortic valve disease and 30 healthy volunteers were analysed. As ECG criteria for LVH, we assessed the Sokolow-Lyon voltage/product, Gubner-Ungerleider voltage, Cornell voltage/product, Perugia-score and Romhilt-Estes score.

**Results:**

All ECG criteria demonstrated a significant correlation with LV mass and chamber size. The highest predictive values were achieved by the Romhilt-Estes score 4 points with a sensitivity of 86% and specificity of 81%. There was no difference in all ECG criteria between concentric and eccentric LVH. However, the intrinsicoid deflection (V6 37 ± 1.0 ms vs. 43 ± 1.6 ms, p < 0.05) was shorter in concentric LVH than in eccentric LVH and amplitudes of ST-segment (V5 -0.06 ± 0.01 vs. -0.02 ± 0.01) and T-wave (V5 -0.03 ± 0.04 vs. 0.18 ± 0.05) in the anterolateral leads (p < 0.05) were deeper.

**Conclusion:**

By calibration with CMR, a wide range of predictive values was found for the various ECG criteria for LVH with the most favourable results for the Romhilt-Estes score. As electrocardiographic correlate for concentric LVH as compared with eccentric LVH, a shorter intrinsicoid deflection and a significant ST-segment and T-wave depression in the anterolateral leads was noted.

## Background

Left ventricular hypertrophy (LVH) is a hallmark of chronic pressure or volume overload of the left ventricle and is associated with a markedly elevated risk of cardiovascular morbidity and mortality. Morphologically, LVH may be characterized by increased wall thickness (concentric LVH), increased chamber volume (eccentric LVH) or both [1,2]. In order to identify LVH, the ECG is widely used as a primary screening tool. Various ECG criteria have been put forward, but there is little information as to the predictive values of the respective criteria for the correct diagnosis. Most importantly, the clinical utility of ECG has been limited by a low sensitivity at quite high specificity. Further, there is limited understanding of the contribution and importance of left ventricular volume, left ventricular mass and the ratio of left ventricular mass to volume on the value and performance of the individual criteria. Previously, the validation of the ECG criteria was mostly based on M-mode or 2D echocardiography for estimation of left ventricular mass (LVM) [3-5]. However, today there is no doubt that cardiovascular magnetic resonance (CMR) is a more accurate and reproducible tool to quantify LVM because of the excellent visibility and the lack of geometric assumptions [6,7]. Therefore, CMR is currently deemed the gold standard for in-vivo measurements of LV mass.

The aim of our study was to evaluate and compare the predictive values of several well-established ECG- criteria for LVH against left ventricular mass and volume as assessed by CMR in a large set of patients with a high prevalence of LVH due to aortic valve disease. Furthermore, we aimed to study the effect of the different geometric forms of LVH (concentric and eccentric) on depolarization and repolarization. To address this issue, we studied 120 patients with aortic valve disease and 30 healthy volunteers without history or evidence of cardiovascular disease.

## Methods

### Patients

The study group consisted of 120 patients (78 men, mean age 59 ± 15 years, 42 women, mean age 65 ± 15 years) who were studied for suspected aortic valve disease. The mean age was 61 ± 15 years (range 22 to 85 years). Among the patients with aortic valve disease 13 (11%) were in atrial fibrillation. The results of a subset of these patients regarding the accuracy of CMR for anatomic planimetry of aortic valve area was reported previously [8].

Additionally, a total of 30 healthy volunteers (CTRL) (13 males and 17 females) with a mean age of 40 ± 12 years (range 19 to 66 years) were assessed by CMR. All volunteers had normal blood pressure and sinus rhythm and no symptoms or history of cardiovascular disease or diabetes.

Written informed consent was obtained from all patients in accordance with requirements of the local institutional ethics committee.

### ECG

A 12-lead standard ECG (10 mm = 1 mV, 50 mm/s) was acquired in supine position during quiet respiration. Subjects with complete bundle branch block were excluded from the analysis. The time interval between ECG and CMR was 3 ± 4 days (range -15 days to 17 days).

For the present analysis, seven ECG criteria for LVH were evaluated from all ECGs (30 controls, 120 patients with aortic valve disease). The criteria are listed in Table 1 and include Sokolow-Lyon voltage (sum of amplitude of the S wave in lead V1 and the R wave in lead V5 or V6 ≥ 3.5 mV) [9] and the Sokolow-Lyon product [4,10]. The Cornell voltage of RaVL + SV3 ≥ 2.8 mV for men and ≥ 2.0 mV for women [11], a Cornell voltage product of (RaVL + SV3) × QRS ≥ 244.0 mVms for men and (RaVL + SV3 + 0.8 mV) × QRS ≥ 244.0 mVms for women [4,10]. The Gubner-Ungerleider voltage was calculated as the sum of amplitude of the R wave in lead I and S wave in lead III ≥ 2.0 mV [2]. The Romhilt-Estes Score was calculated using scores of 5 points (5p) for definite diagnoses LVH or 4 points (4p) for probable diagnoses LVH [12]. The Perugia score was positive in the presence of one or more of the following criteria: SV3 + RaVL >2.4 mV and/or left ventricular strain and/or Romhilt-Estes score of 5 or more points [3].

**Table 1:** Summary of various ecg criteria used for evaluation of lvh

Reference	Formula		Definition of LVH
Sokolow-Lyon Voltage [9]	SV1 + RV5 or RV6		≥3.5 mV

Sokolow-Lyon Product [10]	(SV1 + RV or RV6) * QRS		≥371.0 mVms

Gubner-Ungerleider [2]	RI + SIII		≥2.0 mV

Cornell Voltage [11]	RaVL + SV3		≥2.8 mV (men) ≥2.0 mV (women)

Cornell Voltage Product [10]	(RaVL + SV3) × QRS duration (men) (RaVL + SV3 + 0.8 mV) × QRS duration (women)		≥244.0 mVms

Romhilt-Estes score [12]	1. Amplitude = R or S wave in limb leads ≥2.0 mV or SV1-2 ≥3.0 mV or RV5-6 ≥3.0 mV	3 points	≥5 points: definite LVH ≥4 points: probable LVH
	2. ST-T segment pattern =		
	without digitalis	3 points	
	with digitalis	1 point	
	3. Left atrial involment	3 points	
			
	4. Left axis deviation ≥-30°	2 points	
			
	5. QRS duration ≥0.09 sec	1 point	
			
	6 Intrinsicoid deflection ≥0.05 sec in V5-V6	1 point	

Perugia score [3]	SV3 + RaVL >2.4 mV (men) SV3 + RaVL >2.0 mV (women) And/or Typical strain pattern And/or Romhilt-Estes Score ≥5 points		At least one criterion

### Imaging Methods

CMR studies were performed in supine position on a 1.5 Tesla Siemens MRI Sonata system (Siemens Medical Solutions, Erlangen, Germany) with a phased-array receiver coil and breath-hold acquisitions prospectively gated to the ECG. Cine images were acquired in multiple short axis and long axis views with fast imaging with steady-state-free-precession (SSFP, trueFISP; slice thickness 8 mm, 2 mm gap, echo time 1.53 ms, pixel bandwidth 1.085 Hz, repetition time 3.14 ms leading to a temporal resolution of 43 ms, matrix 256*202).

Image analysis was performed off-line using the semiautomatic ARGUS evaluation program (Siemens Medical Solutions, Erlangen, Germany), which is a part of the commercially available cardiac package of the scanner software. Semiautomated tracking of the endocardial and epicardial borders of short-axis slices was performed. As previously reported, the most basal section was defined as the section in which the left ventricular myocardium extended over at least 50% of the circumference on the enddiastolic and endsystolic images [13]. The apical slice was defined as the final slice showing intracavity blood pool at both enddiastole and endsystole. LVM was measured at end-diastole, which was defined at the beginning of the QRS complex as the frame with largest intraventricular area. LVM was calculated at end-diastole after additional detection of epicardial borders of the LV by subtraction of endocardial volume from epicardial volume multiplied by the specific gravity of myocardium (1.05 g/cm^3^). The LV ejection fraction (EF) was calculated as (LVEDV – LVESV)/LVEDV. The LVM and LVEDV were indexed to body surface area. The ratio of LVMI and LVEDVI (M/V) was used as an indicator of LV remodeling. The classification as concentric or eccentric hypertrophy was based on the gender-specific 95^th ^percentile for LVMI (men, 76 g/m^2^; women, 67 g/m^2^) and M/V ratio (men, 1.12 g/ml; women, 1.14 g/ml) of the control group [14]. An increase in both M/V ratio and LVMI was defined as concentric hypertrophy and a normal M/V ratio with an increased LVMI was defined as eccentric hypertrophy. An increase in M/V and a normal LVMI was defined as concentric remodelling.

### Statistics

Data are presented as mean and SD unless stated otherwise. Group differences were assessed by Student's t-test. Elevated LVM index were defined for men and women separately, based on the reference corresponding to the 95^th ^percentile from the normal volunteers. Relationships between ECG criteria and LVM, LVM index, LVEDV and LVEDV index were assessed by correlation analysis. Spearman's rank correlation method was used to assess the association between discrete ECG criteria and continuous measurements. Logistic regression analysis with forward selection was used to assess the influence of age, gender and the various ECG criteria on LVH. For measurements of sensitivity, specificity, positive predictive value (PPV), negative predictive value (NPV) and accuracy, LVH as defined by CMR was the reference standard against which the performance of ECG criteria was compared for all study subjects with aortic valve disease and normal controls. Finally, receiver-operating-characteristics (ROC) analysis was carried out to calculate the area under the curve (AUC) and to compare the diagnostic performance of the various ECG criteria. The results were analyzed using SPSS software version 12.0 (SPSS Inc.) and MedCalc version 9.3.2 (MedCalc Software, Mariakerke, Belgium). A p-value < 0.05 was considered statistically significant.

## Results

### Patient characteristics: LV structure and LVH prevalence by CMR

Table 2 depicts the CMR characteristics of the 120 patients and 30 volunteers examined in the present study. As compared to CTRL, 86% of the patients demonstrated an elevated LVMI and LVEDVI. An elevated M/V ratio was observed in 58% of the patients. Regarding the geometric patterns of the left ventricle, 11% of the patients displayed normal LV geometry, 4% displayed a concentric remodelling, 49% a concentric LVH and 36% an eccentric LVH.

**Table 2:** CMR characteristics in the study population

	**CTRL**	**Patients**				
		All	Normal LV geometry	Concentric Remodeling	Concentric LVH	Eccentric LVH
N (%)	30	120 (100)	10 (8)	8 (7)	59 (49)	43 (36)
Predominant AS (%)	-	87 (100)	10 (12)	8 (9)	53 (61)	16 (18)
Predominant AR (%)	-	33 (100)	0 (0)	0 (0)	9 (27)	24 (73)
EF (%)	57 ± 6	57 ± 15	55 ± 18	69 ± 9	60 ± 14	50 ± 15*
LVM (g)	97 ± 26	199 ± 72^§^	120 ± 27	120 ± 22	224 ± 77	199 ± 54
LVEDV (ml)	133 ± 24	168 ± 74^§^	131 ± 51	81 ± 13	144 ± 51	226 ± 72*
LVMI (g/m^2^)	51 ± 10	104 ± 33^§^	64 ± 11	64 ± 5	114 ± 34	106 ± 26
LVEDVI (ml/m^2^)	72 ± 10	88 ± 38^§^	69 ± 21	44 ± 7	73 ± 23	120 ± 37*
M/V ratio (g/ml)	0.76 ± 0.18	1.30 ± 0.45^§^	0.95 ± 0.16	1.48 ± 0.31	1.61 ± 0.38	0.92 ± 0.19*

Data are presented as mean ± SD. AS, aortic stenosis; AR, aortic regurgitation; EF, ejection fraction; LVM, left ventricular mass; LVEDV, left ventricular enddiastolic volume; LVMI, left ventricular mass index; LVEDVI, left ventricular enddiastolic volume index; M/V, mass-volume ratio LVM/LVEDV; §, p < 0.05 all patients versus CTRL; *, p < 0.05 concentric LVH versus eccentric LVH.

### LVH prevalence by ECG and correlation between ECG scores and LV structure

The prevalence of LV hypertrophy according to ECG varied markedly across the different criteria, ranging from 33% for Gubner/Ungerleider, 45% for Sokolow-Lyon product, 49% for Cornell voltage, 52% for Sokolow-Lyon voltage, 53% for Cornell product, 61% for Romhilt-Estes Score 5 points, 76% for Perugia score to 80% for Romhilt-Estes score 4 points. The correlation coefficients between the various ECG-LVH parameters and LV mass and size are displayed in Table 3. All ECG criteria for LVH correlated significantly (p < 0.05) but moderately with LVM, LVMI, LVEDV and LVEDVI with the closest association between the Sokolow-Lyon product and LVM, LVEDV, LVMI and LVEDVI. In a multivariate analysis Sokolow-Lyon voltage (partial r^2 ^= 0.16, p = 0.049), Sokolow-Lyon product (partial r^2 ^= 0.22, p = 0.007) and Romhilt-Estes score (partial r^2 ^= 0.19, p = 0.021) remained independent predictors of indexed LVM. None of the ECG criteria was an independent predictor of LVEDVI and M/V in multivariate analysis.

**Table 3:** Correlation coefficients between various electrocardiographic criteria and CMR parameters

	**LVM (g)**		**LVEDV (ml)**		**LVMI (g/m^**2**^)**		**LVEDVI (ml/m^**2**^)**		**M/V (g/ml)**	
	**r**	**p**	**r**	**p**	**r**	**p**	**r**	**p**	**r**	**p**

**Sokolow-Lyon voltage**	0.49	<0.001	0.38	<0.001	0.57	<0.001	0.42	<0.001	0.16	<0.05
**Sokolow-Lyon product**	0.60	<0.001	0.47	<0.001	0.66	<0.001	0.49	<0.001	0.19	<0.05
**Gubner-Ungerleider**	0.32	<0.001	0.20	<0.05	0.33	<0.001	0.18	<0.05	0.21	<0.01
**Cornell voltage**	0.37	<0.001	0.20	<0.05	0.47	<0.001	0.25	<0.01	0.25	<0.01
**Cornell product**	0.44	<0.001	0.28	<0.01	0.53	<0.001	0.32	<0.001	0.24	<0.01
**Romhilt-Estes score**	0.57	<0.001	0.33	<0.001	0.60	<0.001	0.31	<0.001	0.30	<0.001

LVM, LV mass; LVEDV, LV enddiastolic volume; LVM, left ventricular mass index; LVEDVI, left ventricular enddiastolic volume index; M/V, LVMI/LVEDVI ratio

When Sokolow-Lyon voltage, Sokolow-Lyon product, Cornell voltage, Cornell product, Gubner-Ungerleider, Perugia score, Romhilt-Estes score 5 points and Romhilt-Estes score 4 points were entered into a multiple regression analysis together with age and gender, Romhilt-Estes score 4 points remained the strongest predictor of LVH with an odds ratio of 1.82 (CI 1.51–2.20, p < 0.0001).

**Figure 1:**
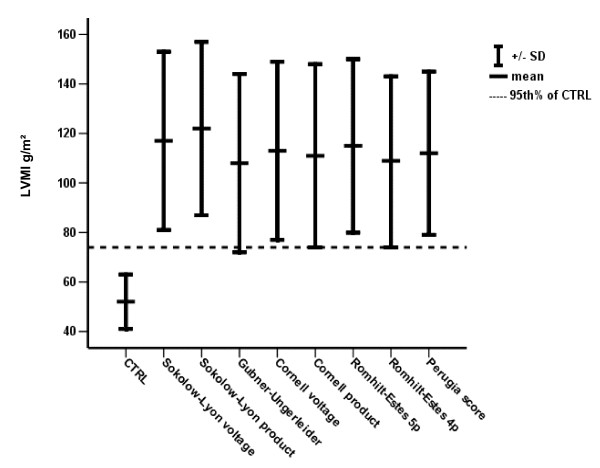
**Varying overlap between 95^th ^percentile of CTRL and mean-SD of positive ECG criteria**.

The mean LVMI of patients with ECG scores indicative of LVH varied between 108 g/m^2 ^and 122 g/m^2 ^according to the utilized ECG score. Of note, the 95^th ^percentile of LVMI of the normal group was within 1 SD of the mean only for Gubner-Ungerleider, Cornell and Romhilt-Estes score 4 points while no overlap was present with the other scores (Figure 1).

### Predictive values of ECG for LVH

**Figure 2:**
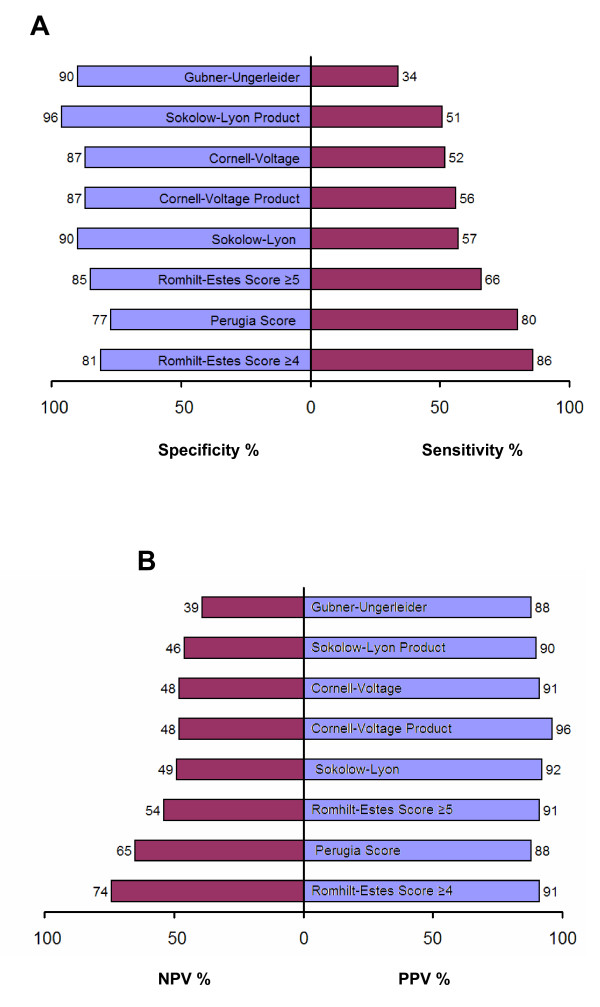
**Sensitivities and specificities (A) and positive predictive values and negative predictive values (B) of the various ECG criteria for LVH determined by CMR**.

The predictive values of the various ECG criteria were computed using the established partition values (Table 4, Figure 2). Of notice, a large variability regarding the predictive values was observed. The Romhilt-Estes score using ≥4 points as cut-off value provided the overall best results with a sensitivity of 86%, a specificity of 81% and an accuracy of 85% in this population group. Comparison of ROC-curves showed a significantly superior better performance of the Romhilt-Estes score as compared to Cornell voltage (p = 0.041), Gubner-Ungerleider (p = 0.001) and Sokolow-Lyon voltage (p = 0.004). No significant differences in ROC-curves were present between Romhilt-Estes score, Cornell product and Sokolow-Lyon product.

**Table 4:** ROC-analysis and predicitve values for ECG-LVH criteria to detect CMR-LVH

	**ROC-AUC (95%-CI)**	**Sensitivity (%)**	**Specificity (%)**	**PPV (%)**	**NPV (%)**	**ACC (%)**
**Gubner-Ungerleider**	0.72 (0.64 – 0.81)	34	90	88	39	52
**Sokolow-Lyon product**	0.86 (0.79 – 0.92)	51	96	90	46	65
**Cornell voltage**	0.78 (0.70 – 0.86)	52	87	91	48	63
**Cornell product**	0.81 (0.73 – 0.89)	56	87	96	48	66
**Sokolow-Lyon voltage**	0.80 (0.73 – 0.88)	57	90	92	49	67
**Romhilt-Estes **	0.87 (0.80 – 0.94)	n.a.	n.a.	n.a.	n.a.	n.a.
**Romhilt-Estes 5p**	n.a.	66	85	91	54	72
**Romhilt-Estes 4p**	n.a.	86	81	91	74	85
**Perugia score**	n.a.	80	77	88	65	79

ROC, receiver-operating-characteristics; AUC, area under curve; CI, confidence interval; PPV, positive predictive value; NPV, negative predictive value; ACC, accuracy; n.a., not applicable

### Comparison eccentric vs. concentric LVH

**Figure 3:**
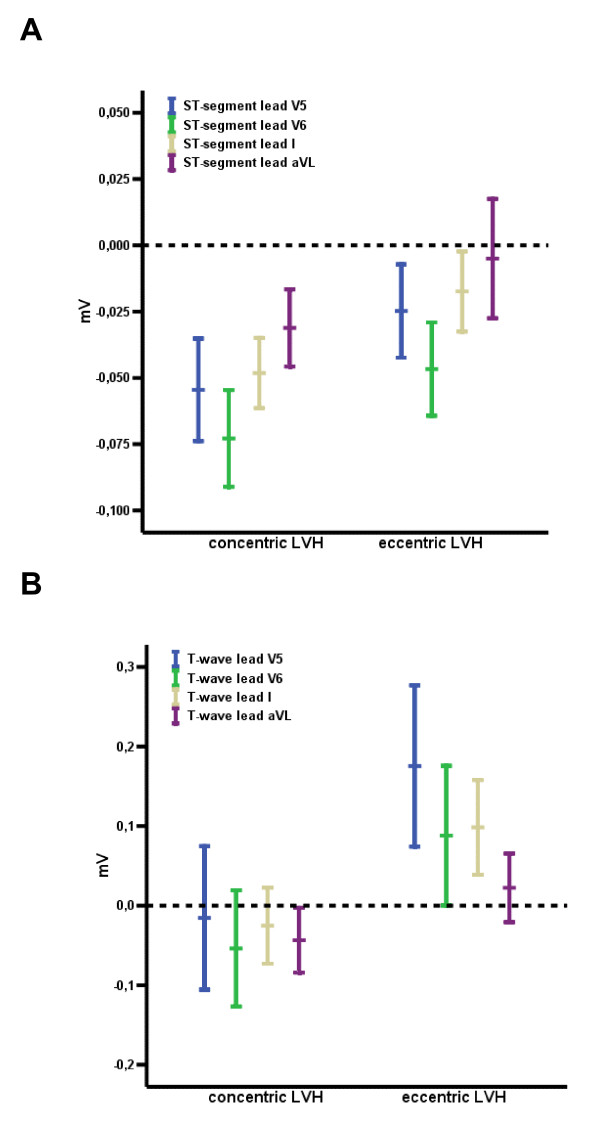
**Significantly decreased ST-segment (A) and T-wave (B) amplitude in leads V5, V6, I and aVL between concentric and eccentric LVH (p < 0.05 for all)**.

**Figure 4:**
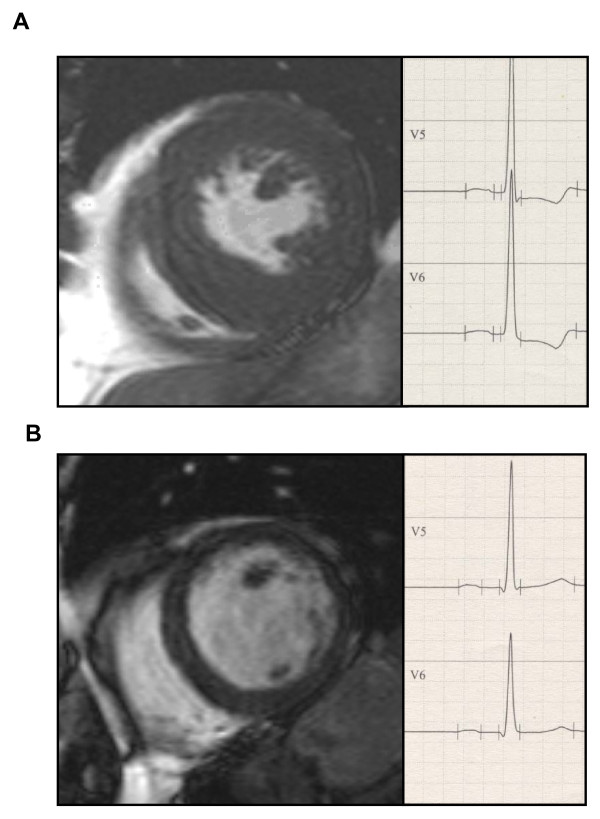
**Representative CMR image and ECG of LV with (A) concentric (LV enddiastolic diameter 42 mm) and (B) eccentric LVH (LV enddiastolic diameter 60 mm)**.

There were no significant differences for ECG criteria between the concentric and eccentric geometry pattern. Accordingly, the predictive values of the different ECG-LVH criteria did not differ for the detection of eccentric versus concentric LVH. Of note, however, depolarization analysis resulted in significant differences of intrinsicoid deflection (V6, 37 ± 1.0 ms in concentric LVH vs. 43 ± 1.6 ms in eccentric LVH, p < 0.01) by no significant differences in QRS duration (101 ± 9 ms in concentric LVH vs.102 ± 8 ms in eccentric LVH, p = 0.60). Furthermore, significant differences were observed regarding ST segments, which were significantly deeper in concentric LVH as compared to eccentric LVH (Figure 3 and 4).

## Discussion

This is the first assessment of the performance of various ECG criteria for identification of LV hypertrophy and enlargement as determined by CMR in a large sample of patients with aortic valve disease. On a second objective, we investigated the effect of concentric and eccentric LVH on depolarization and repolarization.

We found that all ECG criteria correlated significantly with LV mass and chamber size. All ECG criteria reached satisfactory predictive values for the detection of LVH. Of note, the highest predictive values were obtained by the less well known Romhilt-Estes score 4 points which performed better than the widely known Sokolow-Lyon index as well as the other ECG criteria. There were no differences between all ECG criteria regarding the detection of concentric or eccentric LVH. However, intrinsicoid deflection was shorter in concentric LVH and amplitudes of ST-segment and T-wave in the anterolateral leads were deeper.

### LV mass determined by CMR

The results we report in this study were obtained using a SSFP-sequence CMR technique. SSFP-sequence is a modern CMR sequence and has been shown to provide a better contrast between blood pool and endocardial border in comparison to the older turbo-gradient-echo sequence [15]. The turbo-gradient-echo sequence has been demonstrated to overestimate LV mass by 13 g/m^2 ^in comparison to the more recently developed SSFP [16]. Therefore, our normal values, which are very similar to other recently published studies utilizing SSFP sequences, are not comparable with data where a turbo-gradient-echo CMR sequence was utilized [17].

### ECG for diagnosis of LV hypertrophy

In this study, we demonstrate that the Romhilt-Estes score correlates well with LV structure and mass and provides the best overall predictive values for the diagnosis of LVH in patients with aortic valve disease. The Romhilt-Estes score is a unique composite ECG criterion which includes not only QRS-complex amplitude but also the ST-T segment. Probable LVH is defined as 4 or more of 13 possible points. Of note, 3 points will be given if the typical ST-T pattern of left ventricular strain is present without use of digitalis. Or rather, patients with ST-T segment abnormalities tend to be diagnosed as "positive" by Romhilt-Estes score. In the present study, ST-T abnormalities were commonly observed in patients with LVH by aortic valve disease as compared with much lower prevalence in hypertensive patients with LVH [18,19], which could have contributed to a high sensitivity for the Romhilt-Estes score.

The Sokolow-Lyon index is possibly the most widely utilized ECG score for LVH. In the current study the Sokolow-Lyon index also displayed satisfactory correlations with cardiac structural parameters and LV mass. However, the sensitivity and NPV for the Sokolow-Lyon index were rather weak whereas specificity and PPV were rather high. This may be due to the rather high LVMI of patients with positive Sokolow-Lyon index in comparison to the LVMI of normal subjects as depicted in Figure 1. In order to improve sensitivity and NPV of the Sokolow-Lyon index, a reduction of the voltage cutpoint to below 3.5 mV would be necessary.

As a general conclusion from all evaluated ECG scores our data indicate that in patients with aortic valve disease, Gubner-Ungerleider, Sokolow-Lyon and Cornell voltage show a higher specificity and poorer sensitivity compared with Perugia and Romhilt-Estes score. Importantly, the type and extent of LVH may influence the predictive values and our current patients represent mostly moderate or severe LVH due to aortic valve disease. Other studies focused on patients with hypertension, usually with only modest LVH [20,21]. This may explain the finding that an ECG-criteria that performed well in the present study, such as the Romhilt-Estes score, performed less well in other studies [4,20].

### Geometric patterns: effects on depolarization and repolarization

All ECG criteria correlated significantly with LVM, LVMI, LVEDV and LVEDVI, suggesting that LV wall mass and LV cavity size are both responsible for the ECG characteristics typical for LVH. In a descending order, the closest correlation was present for LVMI, then LV cavity size and then for M/V ratio. The relative importance of these parameters for the ECG criteria has not been previously examined. In the current study population, concentric remodelling and concentric LVH was present in approximately one half of the patients. However, no significant difference regarding the predictive values was observed between concentric and eccentric LVH.

Nevertheless, a significant difference in repolarization between patients with concentric and eccentric LVH was observed with less ST-segment depression and more positive T-waves in the anterolateral leads for eccentric versus concentric LVH. Already Cabrera and Monroy first proposed that patients with either pressure or volume overload due to aortic valve disease display differences in repolarization [22]. Since then, a controversy persists and the concept has failed to gain general acceptance because of the wide overlap observed in previous studies. Nevertheless, our current study confirms Cabrera et al. and the ST segment depression and T-wave inversion are statistically significant in concentric as compared to eccentric LVH. In addition, a significant shorter intrinsicoid deflection was observed in concentric versus eccentric LVH.

### Limitations

The current study only evaluated patients with LVH due to aortic valve disease. Hence, our results cannot be extrapolated to patients with LVH secondary to other conditions and predictive values of the ECG LVH criteria may vary depending on the studied cohort. However, due to the lower prevalence of LVH in hypertension, a much larger patient cohort would be necessary to achieve significant results.

## Conclusion

Upon evaluation of several ECG scores for LVH by CMR a wide range of predictive values was observed. The most favourable predictive values were achieved by the Romhilt-Estes score which should therefore be the ECG score of choice for the assessment of LVH in patients with suspected or known aortic valve disease. Additionally, a significantly shorter intrinscoid deflection and a significant ST-segment and T-wave depression in the anterolateral leads were observed as electrocardiographic correlate for concentric as compared to eccentric LVH.

## Competing interests

The authors declare that they have no competing interests.

## Authors' contributions

SB and KD conceived the study with support from GAJR, SF and AL. SB and JH collected data and analysed the data with support from AL. BD, FP and KD oversaw CMR acquisition and analysis. All authors contributed to the preparation of the manuscript, and read and approved the final manuscript.
